# Distribution, lipophilicity and tissue half-life as key factors in sulphonamide clearance from porcine tissues

**DOI:** 10.2478/jvetres-2025-0002

**Published:** 2025-01-30

**Authors:** Artur Burmańczuk, Monika Osypiuk, Bożena Polska, Dominik Kunicki, Marcin Kocik, Karol Grzęda, Włodzimierz Markiewicz, Oktay Yilmaz, Tomasz Grabowski

**Affiliations:** Department of Pharmacology, Toxicology and Environmental Protection, University of Life Sciences in Lublin, 20-033 Lublin, Poland; Private veterinary practice, 33-100 Tarnów, Poland; Private veterinary practice, 24-100 Puławy, Poland; Department of Pharmacology and Toxicology, Faculty of Veterinary Medicine, University of Warmia and Mazury, 10-718 Olsztyn, Poland; Department of Obstetrics and Gynaecology, Faculty of Veterinary Medicine, Afyon Kocatepe University, ANS Campus, 03200 Afyonkarahisar, Türkiye; Department of Inorganic Chemistry, Medical University of Gdańsk, 80-416 Gdańsk, Poland

**Keywords:** sulphonamides, pigs, lipophilicity, Vd, residues

## Abstract

**Introduction:**

Sulphonamides are some of the most widely used antimicrobial drugs in the treatment of bacterial diseases in pigs. The study was conducted to compare the total exposure of tissue to a group of active substances in a single formulation and evaluate the impact of the volume of distribution, lipophilicity and tissue half-life of sulfadimethoxine, sulphathiazole, sulphamethazine and sulphacetamide in two different veterinary drug formulations – Polisulfalent, which was preparation A, and Polisulfamid, which was preparation B.

**Material and Methods:**

Each tested therapeutic preparation was administered to 15 piglets of the Polish Landrace breed. To assume general exposure expressed as the sum of all observed concentrations of all active substances, semi-log regression analyses were performed for both formulations.

**Results:**

The estimated tissue half-life was 21.19 h for preparation A and 17.36 h for preparation B. The comparison of the semi-log regression parameters shows that formulation B’s Y-intercept and slope values were higher than formulation A’s. The upper-bound CI for the Y-intercept and slope value of formulation B was higher than those of formulation A, at 38.7 and 20.8%, respectively.

The lower-bound CI for the Y-intercept and slope value of formulation B was also higher than those of formulation A, at 20.6 and 42.9%, respectively.

**Conclusion:**

Current observations lead to the conclusion that in late-stage drug elimination, it is not the blood half-life, but rather a drug’s volume of distribution which is key in determining the clearance period, this volume being dependent on the physicochemical characteristics of the drug.

## Introduction

Sulphonamides are sulphanilamide subsidiaries, and chemically they are amides of p-amino benzenesulphonic acid (1). The presence of antimicrobial agents in edible tissues of food-producing animals remains a major public health concern, because of the major threat to health posed globally by rising antimicrobial resistance (1, 6, 10, 11, 13, 14, 16, 19). Antibiotics are essential and are widely used in veterinary medicine for therapeutic purposes. One of the challenges is food contamination from veterinary drugs (10, 11, 18, 19, 42, 46). These residues accumulate in animal tissues in manners dependent on the particular drug’s physicochemical properties and pharmacokinetic parameters (3, 4, 5, 10, 44). Among the causes of veterinary drug contamination of food, not accommodating withdrawal periods, not maintaining medical treatment records, overdosing and using unauthorised drugs are all notable (2, 13, 17, 20). Sulphonamides are some of the antimicrobial drugs most widely used to treat bacterial diseases in pigs because of their high efficiency and cost-effectiveness (13, 18, 26, 29). The widespread use of antibiotics in veterinary practice makes the monitoring of residues prudent not only in food, but also in the environment (1, 10, 11, 19, 31, 32). Human intake of sulphonamide residues through foods over time leads to adverse effects, allergies, poisoning and even cancer (4). New regulations have expanded the restrictions on the use of certain antibiotics in animals to a total ban on particular preparations (10, 13, 17, 18, 34). Sulphonamides have been classified in category D (for use with caution) (11, 12). Antibiotics listed in category D, as well as in other categories, must not be used unnecessarily, for too long a period of treatment or in too small a dose (12). Its noted characteristics and permissibility for use make the sulphonamide group a potential first-choice preparation for veterinarians in the treatment of swine diseases (7). The current maximum residue level (MRL) according to EU Regulation 37/2010 is 100 μg/kg (in muscle, fat, liver and kidneys) for all sulphonamides (parent drugs) and in all food-producing species. The same level was also set for bovine, ovine and caprine milk.

The study was conducted to compare the total exposure of tissue to a group of active substances in a single formulation and the impact of the volume of distribution, lipophilicity and tissue half-life of sulphadimethoxine, sulphathiazole, sulphamethazine and sulphacetamide in two different drug formulations.

## Material and Methods

Preparations with the brand names Polisulfalent and Polisulfamid (batch 020307 in both cases; Biowet-Puławy, Puławy, Poland) were used. Polisulfalent and Polisulfamid, respectively formulation A and formulation B, are provided in the form of solutions for injection into horses, cattle, calves, pigs, sheep and dogs. Formulation A consisted of sulphathiazole, sulphadimidine and sulfadimethoxine at 18, 30 and 77 mg/mL, respectively. Formulation B consisted of sulphathiazole, sulphadimidine and sulphacetamide at 30, 50 and 40 mg/mL. Each therapeutic preparation was administered to 15 mixed male and female piglets of the Polish Landrace breed. The subjects were randomly selected, had initial body weight (b.w.) in the range of 12–20 kg and were aged 7–12 weeks. The piglets had water *ad libitum* and were given a well-balanced, specially prepared mixture of cereal meal without antibiotics or growth promoters (AGROPOL concentrates and feed mixtures, Marynin, Poland). These groups were kept in separate metal cages during acclimatisation and the experiment. Each pen with a particular test group was marked accordingly and a numbered ear tag was placed on each piglet for identification. During a seven-day quarantine, the piglets were observed daily for behaviour and health, and a clinical examination was conducted. All animals were qualified for inclusion in further research. One group of pigs was given formulation A and the other formulation B intramuscularly for six days. The initial dose was 1.0 mL/kg b.w., and subsequent ones were half of the initial dose, *i.e*. 0.5 mL/kg b.w. Each formulation group was divided into five subgroups, designated A (day 10), B (day 12), C (day 14), D (day 16) and E (day 18). A subgroup’s numbered day was the slaughter day after the first dose (*e.g*. day 10 was 4 days after the 6-day treatment). Three animals per formulation group were slaughtered on each assigned day of sampling for testing. An analysis method was used which had been validated according to the rules set out in European Commission Decision 2002/657/EC (24, 25, 27, 28, 33, 35, 36). The study was conducted with the authorisation No. 42/2008 of the Local Ethics Committee for Animal Experiments in Lublin, Poland.

For tissue analyses, acetonitrile, methanol, hexane, reverse osmosis–purified water using Milli Q Plus 185 system (Millipore-Waters, Billerica, MA, USA), ammonium acetate, sodium hydroxide, ethyl acetate (all from POCH, Gliwice, Poland unless otherwise stated) and nitrogen were used. High-performance liquid chromatography (HPLC) was performed with a system which comprised a UV-VIS 9050 Detector, STAR 9002 pump and MetaTherm thermostat (Varian Analytical Instruments, Palo Alto, CA, USA), a Synergi 4 μ Fusion-RP 80Å 150 × 4.6 mm column (Phenomenex, Torrance, CA, USA), a nitrogen atmosphere sample evaporation device, a B661S analytical balance (Sartorius, Göttingen, Germany), C18 Oasis hydrophilic–lipophilic balanced (HLB) 1cc solid-phase extraction (SPE) cartridges (Waters, Milford, MA, USA), the Milli Q Plus 185 system and 0.45 μm PTFE syringe filters. The composition of the mobile phase of formulation A consisted of 500 mL of 0.1 M ammonium acetate solution, 330 mL of purified water and 170 mL of acetonitrile, and the mobile phase of formulation B was 870 mL of acetate buffer at pH 4.7 and 130 mL of acetonitrile. Detection was performed at 270 nm.

### Tissue preparation

Muscle, liver, kidney, and fat and skin samples were thawed and dissected into small pieces. Tissue with mass of 1 g was placed into a 10 mL plastic tube with 3 mL of 0.1 M ammonium acetate solution, and 2 mL of hexane was added. The suspension was homogenised for approximately 60 s and centrifuged at 4,000 rpm for 20 min. The organic layer was collected into a clean tube and subjected to SPE. The 30 mg Oasis HLB cartridges were preconditioned with 1 mL of methanol and 1 mL of Milli-Q water. The cartridge was washed with 1 mL of Milli-Q water with 5% methanol and dried for 10 min. The analytes were eluted from the cartridge with 0.5 mL of ethyl acetate and 0.5 mL of acetonitrile. As the final steps, the extract was concentrated to dryness under a nitrogen stream and redissolved with 150 μL of a mixture of mobile phase, and 20 μL of this solution was injected into the HPLC system.

### Method validation

Linearity and precision included the range of 0 to 1,250 ng/g of the concentration of active substances in tissues. Recovery of the sulphonamides from control tissues was evaluated at 1 MRL. The Committee for Veterinary Medicinal Products of the former European Medical Evaluation Agency (the present European Medicines Agency) considered that the sum of all substances belonging to the sulphonamide group in all animal species from which food is obtained should not exceed 100 μg/kg (15). The procedure used allowed us to determine whether the analytical method met the acceptance criteria and allowed us to reliably determine the concentration of sulphonamides in tissues. The quantitative HPLC method was validated for each of the tissues and sulphonamides in terms of linearity, specificity, intra-day and inter-day precision, recovery, decision limit (CC_α_) and detection capacity (CC_β_) according to European Commission Decision 2002/657/EC (9).

The limit of detection (LOD) and the limit of quantification (LOQ) were calculated as

LOD = 3.3·Syx/a and LOQ = 10·Syx/a,

where Syx is the standard deviation and a is the slope.

Repeatability and recovery were assessed by performing tests on tissue blank samples. Twelve samples were fortified at 1 MRL with all the analysed sulphonamides, which meant at 100 ng/g. The analytes were examined in triplicate on three successive days to find the mean concentration, standard deviation and coefficient of variation (%) of the fortified samples.

The CC_α_ was evaluated by analysing 20 blank milk samples fortified with the analyte at the MRL. The concentration at the fortified level plus 1.64 times the corresponding standard deviation equalled the CC_α_. The value of the decision limit plus 1.64 times the corresponding standard deviation equalled the CC_β_.

### Statistical analysis

Determination of the clearance time was based on the method of statistical linear regression analysis including all observed concentrations. The European Medicines Agency’s WT 1.4 software was used for analysis. To assume general exposure expressed as the sum of all observed concentrations and all active substances, semi-log regression analyses were performed using GraphPad 10.0.3 for both formulations (GraphPad Software, San Diego, CA, USA). The best fit was selected based on the Akaike information criterion (AIC). Semi-log analysis was performed based on the equation

Y=Y-intercept + slope ×LogX,

where Y represents concentration and X the time without weighting, considering the mean value of each observation with a 99% confidence interval with identification of unstable parameters.

The half-life of elimination of the sum of sulphonamides from the tissue compartment (t_1/2tissue_) was calculated based on a regression equation. This equation was used for the simulation of concentrations at 10 d and 16 d (exactly 15.9 d, in order to avoid negative values). Natural logarithm values of predicted concentrations at specific time points were used for slope calculation. In the last step, the t_1/2tissue_ was calculated from the formula Ln(2)/slope. Analyses including those of specific tissues and specific drugs in each tissue type were performed with linear regression analysis by GraphPad 10.0.3. Linear regression analysis was performed based on the equation

Y = Y intercept + slope × X, without weighting, considering the mean value of each observation with a 90% confidence interval with the identification of unstable parameters.

The range of analyses covered injection sites, kidneys, the liver, muscle, and fat and skin; the 90% confidence interval for the Y-intercept and slope; the R^2^ coefficient determination; the root mean squared error (RMSE); the Shapiro–Wilk test; and the normality Shapiro–Wilk test. In the current research, clinical significance refers to the practical importance of the effect, indicating whether a drug has a meaningful impact on physiology (23). This concept is distinct from statistical significance, which merely assesses whether an observed effect is likely due to chance. Clinical significance assesses whether a drug leads to a noticeable change in physiology. Clinical significance is often assessed using metrics such as the minimal clinically important difference, which defines the smallest change in a treatment outcome that may be observed. In this publication, clinical significance focuses on the meaningfulness of adverse reactions between drug formulations observed (8).

## Results

The linearity of the method was confirmed to be in the range 0 to 1,250 ng/g of the tissue, and the R^2^ to be >0.999. The LOD and the LOQ for sulphonamides were calculated in the ranges of 0.90–2.89 ng/g and 2.73–8.76 ng/g, respectively. The absence of interfering substances was confirmed based on the analysis of 12 blank matrices per tissue type. No interfering peaks from endogenous compounds nor from other antibacterial agents were found in the retention time of the target sulphonamides. For intra-day and inter-day assays, recoveries were within the acceptance criteria for all examined analytes. The values were >60% at 100 ng/g in all tissues except for sulphacetamide recovery, which was 45% in the case of fat and skin and of liver, and 48% in the case of muscle. The highest recovery, 90%, was noted for sulphamethazine in all tissues and sulphadimethoxine in the liver. The relative standard deviation was below 10%. The recovery of inter-day repeatability values was >62% and the relative standard deviation was below 9%.

The values of the CC_α_ for sulphacetamide in the liver, kidney, muscle, and fat and skin were 55 ng/g, 80 ng/g, 65 ng/g and 51 ng/g, respectively. The values of the CC_β_ were higher and amounted to 57 ng/g, 82 ng/g, 68 ng/g and 52 ng/g, respectively. The values of the CC_α_ and CC_β_ for sulphathiazole were similar in the liver and fat and skin and were 66 ng/g and 68 ng/g, respectively. The CC_α_ for sulphathiazole in kidney and muscle had values of 98 ng/g and 115 ng/g, respectively, and the CC_β_ 96 ng/g and 110 ng/g, respectively. The CC_α_ and CC_β_ for sulphamethazine were similar in the liver, kidney, and fat and skin and were found to be 89 ng/g and 91 ng/g, respectively. They were higher for this sulphonamide in the muscle and were 101 ng/g and 103 ng/g, respectively. The values of the CC_α_ and CC_β_ for sulphadimethoxine were higher in all analysed tissues than for the other sulphonamides. The CC_α_ reached the levels of 129 ng/g, 104 ng/g, 125 ng/g and 120 ng/g in the liver, kidney, muscle, and fat and skin, respectively. Comparably, the values of the CC_β_ were at the levels of 130 ng/g, 106 ng/g, 126 ng/g and 121 ng/g in the liver, kidney, muscle, and fat and skin, respectively. Method performance data concerning the antibiotics studied in porcine tissues are shown in Table 1.

**Table 1.** Performance data for the method detecting antibiotic residues in porcine tissues

Analyte	Tissue	LOD ng/g	LOQ ng/g	CC_α_ ng/g	CC_β_ ng/g	Recovery %	Intra-day precision %	Inter-day precision %
Sulphadimethoxine	Muscle	1.02	3.08	124.57	125.70	74.56	3.49	4.12
	Liver	1.17	3.54	128.60	129.50	96.23	3.21	4.56
	Kidney	1.25	3.78	103.78	105.70	74.78	3.52	5.18
	Fat and skin	0.90	2.73	119.96	120.44	77.34	3.02	5.96
Sulphathiazole	Muscle	1.17	3.55	101.01	106.27	84.41	4.38	3.85
	Liver	1.20	3.65	107.84	109.60	81.49	1.58	4.55
	Kidney	1.28	3.90	110.03	113.72	86.67	9.57	7.12
	Fat and skin	1.06	3.20	100.28	102.82	64.76	4.36	5.47
Sulphamethazine	Muscle	1.34	4.06	101.00	103.00	90.69	5.23	6.11
	Liver	2.72	8.25	85.45	87.91	95.75	2.06	4.23
	Kidney	1.47	4.47	87.53	89,17	90.13	2.61	3.88
	Fat and skin	1.08	3.28	88.52	100.45	92.68	0.95	2.45
Sulphacetamide	Muscle	1.54	4.67	65.23	68.11	48.02	4.05	4.41
	Liver	0.98	2.98	54.89	55.31	45.48	4.18	4.12
	Kidney	1.31	3.97	79.96	82.12	59.99	3.88	4.88
	Fat and skin	2.89	8.76	50.87	52.22	44.96	1.49	3.33

1 LOD – limit of detection; LOQ – limit of quantification; CC_α_ – decision limit; CC_β_ – detection capability

**Fig. 1.**
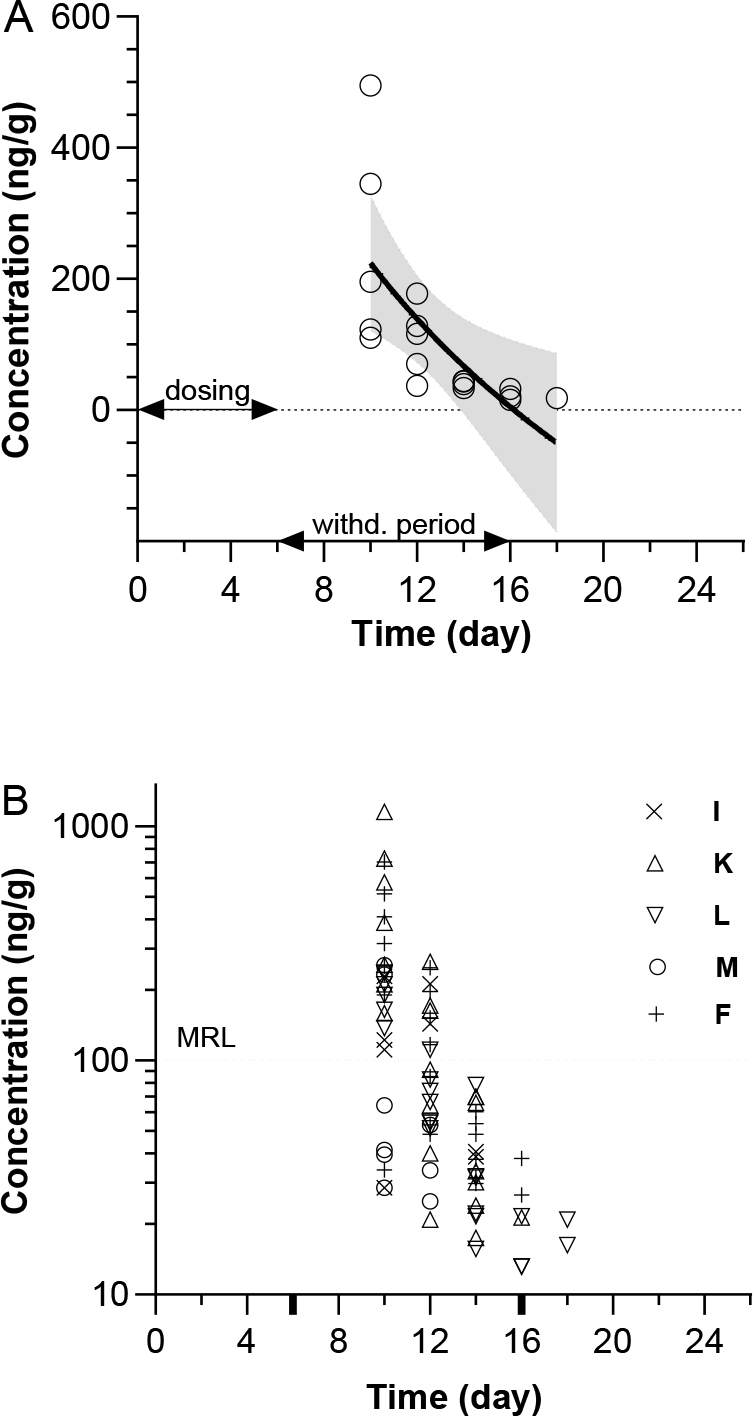
Total exposure to formulation A active substances (sulphathiazole, sulphamethazine and sulphadimethoxine). A – nonlinear semi-log fit of all observed concentrations (black line) with 99% confidence intervals (dashed lines). Circles represent arithmetic means in one tissue type based on observed concentrations of all active substances. B – variability of sulphathiazole, sulphamethazine and sulphadimethoxine observed concentrations per tissue. MRL – maximum residue limit; I – injection site; K – kidneys; L – liver; M – muscle; F – fat and skin. Bold ticks on the time axis represent the in B represent the start and finish of the designed 10-day withdrawal period

Semi-log clearance times of formulation A’s constituents are shown in Fig. 1A. The observed concentration variability across analysed tissues for all active substances in formulation A is shown in Fig. 1B. The calculated Y-intercept and slope values (with 99% CI) were 1,298 (421.5 to 2,175) and −1,074 (−1,867 to −281.1), and the parallel R^2^, RMSE, AIC and Shapiro–Wilk test values were 0.4945, 90.16, 168.7 and 0.8595. With a P-value of 0.0120 in the Shapiro–Wilk test, the observed data passed the normality test at a = 0.01. The t_1/2tissue_ was estimated at 21.19 h.

**Fig. 2.**
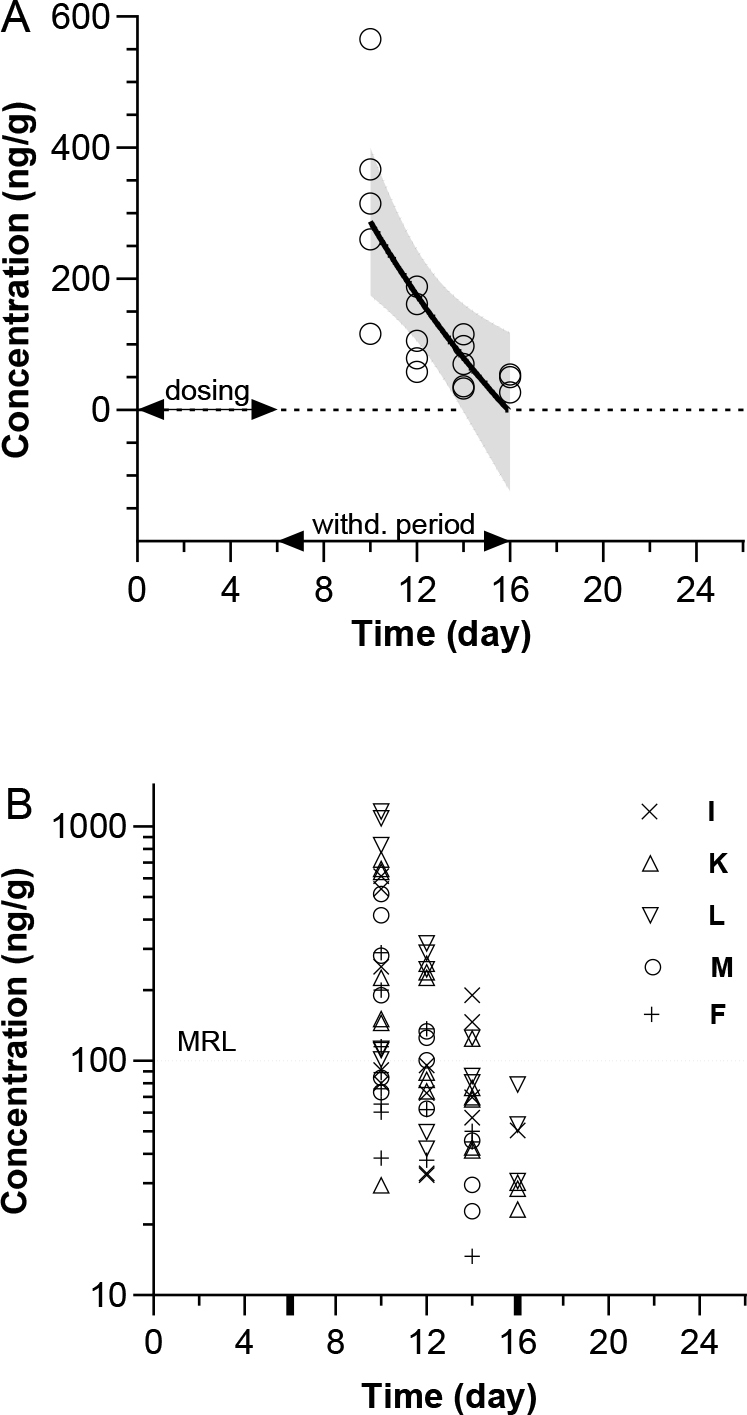
Total exposure to formulation B active substances (sulphathiazole, sulphamethazine and sulphacetamide). A – nonlinear semi-log fit of all observed concentrations (black line) with 99% confidence intervals (dashed lines). Circles represent arithmetic means in one tissue type based on observed concentrations of all active substances. B – variability of sulphathiazole, sulphamethazine, and sulphacetamide concentrations observed in different tissues. MRL – maximum residue limit; I – injection site; K – kidneys; L – liver; M – muscle; F – fat and skin. Bold ticks on the time axis in B represent the start and finish of the 10-day withdrawal period

Semi-log clearance times of formulation B’s constituents are shown in Fig. 2A. The observed concentration variability across analysed tissues for all active substances in formulation B is shown in Fig. 2B. The calculated Y-intercept and slope values (with 99% CI) were 1,713 (687.7 to 2,738) and −1,425 (−2,358 to −492.3), and the parallel R^2^, RMSE, AIC and Shapiro– Wilk test values were 0.5545, 95.55, 170.8 and 0.9096. With a P-value of 0.0846 in the Shapiro–Wilk test, the observed data passed the normality test at a = 0.01. The t_1/2tissue_ was estimated at 17.36 h.

The comparison of the semi-log regression parameters shows that formulation B’s Y-intercept and slope values were higher than formulation A’s. The upper-bound CI for the Y-intercept and slope value of formulation B was higher than those of formulation A, at 38.7 and 20.8%, respectively. The lower-bound CI for the Y-intercept and slope value of formulation B was also higher than those of formulation A, at 20.6 and 42.9%, respectively. The parameters of linear regression representing clearance of the active substances of formulations A and B from each tissue type are presented in Table 1.

Of the linear fittings presented in Table 2, 21 allowed the analysis of Y-intercept and slope values. A total of 21 data series representing different tissues and different active substances were ranked from the highest to the lowest Y-intercept value. Then, the group was divided into three groups of seven observations: H – high Y-intercept values, M – mediate Y-intercept values, and L – low Y-intercept values. For each of the categories, an arithmetic mean was determined. In this way, H, M, and L Y-intercept values equal to 783.38, 493.54 and 240.37, respectively, were obtained. In the same groups, the average value of lipophilicity in the group was also determined. The arithmetic means of the H, M, and L XLogP values were 0.67, 0.3 and 0.03. The correlation coefficient R^2^ for the Y-intercept and XLogP in the analysed categories was 0.997.

**Table 2.** Linear regression fit parameters of all observed concentrations per active substance and tissue

Tissue	Y-Intercept (CI)	Slope (CI)	R^2^	RMSE	W	P-value	Nt
Formulation A							
M1	na	na	na	na	na	na	na
M2	na	5.461 (−4.075e+017– 4.075e+017)	0	114.6	0.79	0.0912	No
M3	534.4 (−268.5–1337)	−41.42 (−114.1–31.26)	0.2695	74.7	0.897	0.3561	Yes
K1	396.7 (−105.7–899.2)	−26.01 (−65.86–13.83)	0.6451	36.95	0.952	0.7292	Yes
K2	923.3 (511.3–1,335)	−66.94 (−101.7–−32.18)	0.7001	73.13	0.964	0.8453	Yes
K3	2,195 (1,351–3,039)	−150.9 (−218.2–−83.71)	0.6854	211.2	0.872	0.1067	Yes
L1	na	na	na	na	na	na	na
L2	409.1 (249.9–568.3)	−25.05 (−37.25–−12.86)	0.646	50.07	0.891	0.1755	Yes
L3	394.1 (276.8–511.3)	−23.76 (−32.51–−15.02)	0.708	40.02	0.965	0.854	Yes
F1	na	na	na	na	na	na	na
F2	659.5 (445.4–873.6)	−43.75 (−60.80–−26.69)	0.7399	53.58	0.976	0.9425	Yes
F3	1,442 (987.5–1897)	−97.05 (−133.3–−60.83)	0.7563	113.8	0.898	0.2076	Yes
I1	na	na	na	na	na	na	na
I2	138.6 (−572.5–849.6)	−4.772 (−65.97–56.42)	0.02527	56.75	0.95	0.7127	Yes
I3	468.9 (−429.0–1,367)	−27.92 (−105.2–49.35)	0.3576	71.67	0.95	0.7139	Yes
Formulation B							
M1	1,320 (899.8–1,740)	−92.89 (−127.5–−58.30)	na	na	na	na	na
M2	417.8 (299.6–535.9)	−26.03 (−35.45–−16.62)	0.8541	76.77	0.929	0.5405	Yes
M4	na	na	0.2053	52.74	0.879	0.336	Yes
K1	1,676 (1,316–2,037)	−109.8 (−137.7–−81.91)	na	na	na	na	na
K2	421 (353.5–488.5)	−31.26 (−37.48–−25.03)	0.8525	102.3	0.902	0.1961	Yes
K4	na	na	0.7678	29.57	0.837	0.0411	No
L1	2,374 (1,718–3,031)	−154.6 (−204.4–−104.8)	na	na	na	na	na
L2	na	4.859 (−9.685e+016– 9.685e+016)	0.7603	202.8	0.937	0.4546	Yes
L4	na	na	0.979	5.018	0.829	0.1371	Yes
F1	544.1 (220.6–867.6)	−37.01 (−63.72–−10.30)	na	na	na	na	na
F2	157 (−15.13–329.1)	−7.13 (−22.11–7.852)	0.4961	64.61	0.967	0.8657	Yes
F4	na	na	na	27.25	0.999	0.9427	Yes
I1	1,037 (401.6–1673)	−67.52 (−118.1–−16.89)	na	na	na	na	na
I2	1,037	−67.52	0.4346	159.1	0.929	0.4364	Yes
I4	na	na	0.9003	5.48	1	>0.9999	Yes

1 I – injection site; K – kidneys; L – liver; M – muscle; F – fat and skin; numbers with tissue letters: 1 – sulphathiazole, 2 – sulphamethazine, 3 – sulphadimethoxine, 4 – sulphacetamide; CI – 90% confidence interval; R^2^ – squared coefficient determination; RMSE – root mean squared error; W – Shapiro–Wilk test; Nt – normality test; N – number of observations; na – not applicable

## Discussion

The present studies show the total exposure to the active components of two different sulphonamide drugs. This paper presents evidence that clearance down to the adopted MRLs does not happen any earlier than before the end of the withdrawal period. The total exposure to the analysed substances was also determined. It was proven that the differences in the composition of the two formulations did not have any clinically significant effect on the active substance concentrations before the end of the withdrawal period.

In the estimation of the risk of the presence of veterinary drugs in animal tissues, it is not only the confirmation of the relationship between withdrawal time and MRL which provokes discussion: it is also open to debate how interactions between different active substances can modify their elimination, and it is also an unresolved question whether the sum of pharmacologically active substances present at levels lower than the MRL may pose a threat to human health.

The analysed formulations contained very similar total amounts of sulphonamides. These were 125 mg in formulation A and 120 mg in formulation B. However, a summary analysis of all observed data shows statistically significant differences in the characteristics of the regression curves (Figs 1A and 2A). The characteristics of the curves do not lead to different parameters for each formulation related to the safety of its use. However, the Polisulfalent and Polisulfamid pair may be an example of a model system in which the components can significantly modify the kinetics of xenobiotic withdrawal in the late elimination phase.

Generalising the observations made, it can be concluded that formulation B was characterised by higher levels of observed concentrations on the 10^th^ day from the first administration of the drug than formulation A. However, this characteristic was accompanied by a much steeper slope of this formulation’s curve, which in effect led to the same rapid clearance of sulphonamides as in the case of formulation A. The main difference between the two formulations is the presence of only one lipophilic ingredient in formulation A, which is sulphadimethoxine (XLogP = 1.6). The other constituents of formulation A and formulation B were only ingredients which were hydrophilic in different amounts (sulphathiazole’s XLogP was 0.1, sulphadimidine’s 0.3 and sulphacetamide’s −1.0). In this case, lipophilicity is reflected by the volume of distribution of individual sulphonamides.

The volume of distribution is cited as an important factor influencing the characteristics of the clearance time of veterinary drugs in the context of its impact on their late elimination (30, 40, 41, 43, 48). While the volume of distribution for sulphadimethoxine is not high and is around 0.25 L/kg, it has been found to be higher in the case of sulphathiazole, sulphadimidine and sulphacetamide and was 1.16, 0.36 and 0.6 L/kg, respectively (39, 42, 45, 47). A low volume of distribution of sulphadimethoxine consequently means a smaller amount of the drug that the organism has to clear from tissues. This lesser need for clearance may explain the significantly lower initial concentrations observed in the current studies for the formulation containing lipophilic sulphadimethoxine. The much larger volumes of distribution of the formulation B components contributed to a much larger pool of molecules that needed to be removed from tissues for total clearance.

The above assumptions and observations lead to the conclusion that in late-stage drug elimination, it is not the blood half-life, but rather a drug’s volume of distribution which is key in determining the clearance period, this volume being dependent on physicochemical characteristics of the drug (21, 22, 36). This hypothesis was partially confirmed by the linear relationship (R^2^ > 0.997) between the Y-intercept and the XLogP value of the analysed sulphonamides. It is interesting to note that an analogous analysis dividing individual drugs and tissues into the three categories H, M and L in terms of regression slope value categorises tissues according to this value to some extent. In category H (rapid elimination from tissue), out of seven tissues, as many as three are tissue from the kidney. In category L (free elimination from tissue), out of seven tissues, again three are the same tissue, this time liver. This is in line with previous research conclusions, as pig liver may contain even more than 5% fat (35).

In analysing the arithmetic mean of the Y-intercept and the slope between tissues, the highest values of Y-intercept were recorded for the kidneys, the next-highest for the liver, somewhat lower values were seen for the muscles, lower ones still for injection sites, and fat and skin had the lowest. No significant correlations between slope and Y-intercept values for individual active substances were confirmed. However, only sulphamethazine was present in as many as 9 out of 21 models, as opposed to sulphathiazole of which the clearance was represented by only 3 tissue models. It is pertinent that in as many as 7 cases the concentrations of sulphathiazole were so low that it was not possible to determine the slope or Y-intercept for the withdrawal process. This formulation-independent observation unequivocally confirms that in the studied group of active substances, sulphathiazole was eliminated from tissues with the highest efficacy.

Based on the elimination kinetics during the withdrawal period, the tissue half-life for the sum of sulphonamides in formulations A and B was also determined. The values obtained support the thesis that both preparations’ volume of distribution was an important withdrawal factor, and that t_1/2tissue_ was another. It should be noted that the estimated t_1/2tissue_ values for formulation B were much shorter than those for formulation A (B t_1/2tissue_ of 17.36 h *versus* A t_1/2tissue_ of 29.11 h). A second tissue half-life observation to draw attention to is that the estimated t_1/2tissue_ was much longer than the t1/2kel value (the elimination half-life determined for blood) because it concerned tissue compartments with completely different physicochemical characteristics to those of the blood compartment. These differences in tissue half-lives are critical for determining appropriate withdrawal periods to ensure that pork is free from harmful residues before entering the food supply.

The confirmed difference in t_1/2tissue_ between the two formulations corresponded perfectly with the differences in the amount of lipophilic components present in the formulation. In the case of formulation A, more than 61% of the active substances was lipophilic sulphadimethoxine, which was not present in formulation B. This is reflected in the difference between t_1/2tissue_ values, over 67% longer being needed for formulation A than formulation B, which only contained hydrophilic constituents. This relationship seems to be logical and results from the much greater potential of lipophilic drugs to be freely distributed and be deposited in tissues inaccessible to hydrophilic drugs. Lipophilicity not only makes it possible to reach compartments such as fat, but also means that these types of compartments or sub-compartments within other tissues can also be accessed. It should be added that redistribution from tissues such as fat is completely different to redistribution from highly vascularised organs, which is because of huge differences between the levels of blood perfusion in fat and in organs. This distinction is crucial, as it influences the clearance times of sulphonamides from these compartments, ultimately affecting the withdrawal periods necessary to ensure residue-free status in pork.

The effect of the metabolism and in particular the hepatic metabolism of lipophilic drugs is also a factor in their clearance times. It is known that as lipophilicity increases, drugs bind more easily to blood plasma proteins. This also means that they have an increasing affinity for various types of other proteins, including metabolic proteins. It would follow that drugs with high lipophilicity should be metabolised faster and thus be in lower concentrations in the blood and, consequently, also in tissues. This logical contention would contradict the current inference regarding formulations A and B. However, it should be taken into account that the deposition of lipophilic drugs in tissues, for which, due to the drugs’ physicochemical characteristics, they have a high affinity, leads to the formation of a kind of depot. A drug bound to a tissue that can be a deep compartment for it is protected from metabolic processes to some extent. Therefore, the long elimination of highly lipophilic drugs from tissues does not have to be associated with high concentrations of these drugs in the bloodstream, because it is the result of a kind of tissue uptake. The human health implications of multiple active substances being present below MRLs in pork may be clinically significant. This is one of the hypotheses that we formed after the current work. This hypothesis seems to be accurate, because people consume many different animal products and each of them may contain different amounts of drugs used in veterinary medicine. In addition, these tissues may contain both herbicides and pesticides. If we assume that, in the worst-case scenario, a person ingests products containing many different substances at levels below the MRL, a scenario may become possible in which the effects of many different substances are potentialised, which could exert a clinically significant effect in combination. Unfortunately, the current regulations and guidelines do not propose either methodological or regulatory solutions that would protect consumers against such a scenario.

The current results indicate both the need for further research and the impact of interactions between drugs and their metabolites on potential risks for consumers. The current state of knowledge allows us to launch such research programmes based on, for example, physiologically based pharmacokinetics models.

## Conclusion

The present studies are limited in scope, but they allow us to build a model-based approach because they analysed the distribution of several chemical homologues in different tissues simultaneously. The key conclusion from the study is the confirmation of an impact on the clearance of drugs from tissues and organs which is proportional to their distribution capabilities and physicochemical characteristics. The conducted studies prove that physicochemical affinity modulates t_1/2tissue_ as a kind of dose substitute and modulates distribution possibilities.

## References

[1] Abdallah H., Arnaudguilhem C., Lobinski R., Jaber F. (2015). A multi-residue analysis of sulphonamides in edible animal tissues using QuEChERS extraction and HPLC-MS/MS. Anal Methods.

[2] Almeida S.A.A., Moreira F.T.C., Heitor A.M., Montenegro M.C.B.S.M., Aguilar G.G., Sales M.G.F. (2011). Sulphonamide-imprinted sol-gel materials as ionophores in potentiometric transduction. Mater Sci Eng C.

[3] Antimicrobial Resistance Collaborators (2022). Global burden of bacterial antimicrobial resistance in 2019: a systematic analysis. Lancet.

[4] Arroyo-Manzanares N., Lara F.J., Airado-Rodriguez D., Gamiz-Gracia L., Garcia-Campana A.M. (2015). Determination of sulfonamides in serum by on-line solid-phase extraction coupled to liquid chromatography with photoinduced fluorescence detection. Talanta.

[5] Burmańczuk A., Milczak A., Grabowski T., Osypiuk M., Kowalski C. (2016). The using of a piglets as a model for evaluating the dipyrone hematological effects. BMC Vet Res.

[6] Burmańczuk A., Roliński Z., Kowalski C., Zań R. (2016). Pharmacokinetic - pharmacodynamic model and ampicillin residue depletion after intramammary administration in cows. J Vet Res.

[7] Chantziaras I., Boyen F., Callens B., Dewulf J. (2014). Correlation between veterinary antimicrobial use and antimicrobial resistance in food-producing: a report on seven countries. J Antimicrob Chemother.

[8] Chen L., Wang X., Lu W., Wu X., Li J. (2016). Molecular imprinting: perspectives and applications. Chem Soc Rev.

[9] Commission of the European Communities (2002). Commission Decision of 12 August 2002 implementing Council Directive 96/23/EC concerning the performance of analytical methods and the interpretation of results (2002/657/EC). OJEC L.

[10] Dibbern D.A.Jr., Montanaro A. (2008). Allergies to sulfonamide antibiotics and sulfur-containing drugs. Ann Allergy Asthma Immunol.

[11] European Commission (2010). Commission Regulation No 37/2010/EC of 22 December 2009 on pharmacologically active substances and their classification regarding maximum residue limits in foodstuffs of animal origin. OJEU L.

[12] European Medicines Agency, Committee for Medical Products for Veterinary Use (CVMP), Committee for Medical Products for Human Use (CHMP) (2019). EMA/CVMP/CHMP/682198/2017-Categorisation of antibiotics in the European Union.

[13] European Medicines Evaluation Agency, Committee for Medical Products for Veterinary Use (CVMP) (1997). EMEA/CVMP/036/95-FINAL Note for Guidance on Approach toward harmonization of withdrawal period.

[14] European Medicines Evaluation Agency, Committee for Medical Products for Veterinary Use (CVMP), Veterinary International Conference on Harmonisation (VICH): EMEA/CVMP/590/98-Final VICH GL1 Validation of analytical procedures (1998). definition and terminology-Scientific guideline and EMEA/CVMP/591/98-Final VICH GL2: Validation of analytical procedures: Methodology-Step 7 consensus guideline.

[15] European Medicines Evaluation Agency, Committee for Veterinary Medical Products (1995). EMEA/026/95 Sulphonamides (2) Summary Report.

[16] European Parliament and the Council of the European Union (2001). Directive 2001/83/EC of the European Parliament and of the Council of 6 November 2001 on the Community code relating to medicinal products for human use. OJ EC L.

[17] (2020). Food and Agriculture Organization of the United Nations.

[18] Food and Drug Administration (2019). United States Code of Federal Regulations Title 21, Part 556, Tolerances for residues of new animal drugs in food.

[19] Fuh M.R.S., Chu S.Y. (2003). Quantitative determination of sulfonamide in meat by solid-phase extraction and capillary electrophoresis. Anal Chim Acta.

[20] Gao R., Zhang J., He X., Chen L., Zhang Y. (2010). Selective extraction of sulfonamides from food by use of silica-coated molecularly imprinted polymer nanospheres. Anal Bioanal Chem.

[21] Grabowski T., Jaroszewski J.J., Feder M., Piotrowski W. (2012). Qualitative structure residue relationship analysis in the determination of the maximum residue limit of veterinary drugs. Chemosphere.

[22] Grabowski T., Jaroszewski J.J., Gad S.C., Feder M. (2012). Correlation between in silico physicochemical characteristics of drugs and their mean residence time in human and dog. Int J Toxicol.

[23] Grabowski T., Tomczyk A., Wolc A., Gad S.C. (2021). Between Biological Relevancy and Statistical Significance – Step for Assessment Harmonization. Am J Biomed Sci Res.

[24] Haupt K. (2003). Imprinted polymers—tailor-made mimics of antibodies and receptors. Chem Commun.

[25] He C., Long Y., Pan J., Li K., Liu F. (2007). Application of molecularly imprinted polymers to solid-phase extraction of analytes from real samples. J Biochem Biophys Methods.

[26] He J., Wang S., Fang G., Zhu H., Zhang Y. (2008). Molecularly imprinted polymer online solid-phase extraction coupled with high-performance liquid chromatography-UV for the determination of three sulfonamides in pork and chicken. J Agric Food Chem.

[27] He J., Zhu Q., Deng Q. (2007). Investigation of imprinting parameters and their recognition nature for quinine-molecularly imprinted polymers. Spectrochim Acta A Mol Biomol Spectrosc.

[28] He X., Tan L., Wu W., Wang J. (2016). Determination of sulfadiazine in eggs using molecularly imprinted solid-phase extraction coupled with high-performance liquid chromatography. J Sep Sci.

[29] Huang Y.H., Xu Y., He Q.H., Cao Y.S., Du B.B. (2012). Determination of sulfadiazine residues in pork by molecular imprinted column coupling with high performance liquid chromatography. Chin J Anal Chem.

[30] Joint FAO/WHO Expert Committee on Food Additives (2004). FAO Food and Nutrition Paper 41/16: Residues of some veterinary drugs in animals and foods. Monograph prepared by the sixty-second meeting of the Joint FAO/WHO Expert Committee on Food Additives Rome.

[31] Kechagia M., Samanidou V., Kabir A., Furton K.G. (2018). One-pot synthesis of a multi-template molecularly imprinted polymer for the extraction of six sulfonamide residues from milk before high-performance liquid chromatography with diode array detection. J Sep Sci.

[32] Kim H.J., Jeong M.H., Park H.J., Kim W.C., Kim J.E. (2016). Development of an immunoaffinity chromatography and HPLC UV method for determination of 16 sulfonamides in feed.. Food Chem.

[33] Kugimiya A., Takei H. (2008). Selectivity and recovery performance of phosphate-selective molecularly imprinted polymer. Anal Chim Acta.

[34] Littlefield N.A., Sheldon W.G., Allen R., Gaylor D.W. (1990). Chronic toxicity/carcinogenicity studies of sulphamethazine in Fischer 344/N rates: two-generation exposure. Food Chem Toxicol.

[35] Liu J., Jiang M., Li G., Xu L., Xie M. (2010). Miniaturized salting-out liquid-liquid extraction of sulfonamides from different matrices. Anal Chim Acta.

[36] Liu Y., Liu X., Wang J. (2003). The adsorption property of nicotine-imprinted polymer. Chin J Anal Chem.

[37] Lyu W., Xiang Y., Wang X., Li J., Yang C., Yang H., Xiao Y. (2022). Differentially Expressed Hepatic Genes Revealed by Transcriptomics in Pigs with Different Liver Lipid Contents. Oxid Med Cell Longev.

[38] Marczak M., Okoniewska K., Okoniewski J., Grabowski T., Jaroszewski J.J. (2015). Indirect relationship between lipophilicity and maximum residue limit of drugs determined for fatty tissue. Bull Vet Inst Pulawy.

[39] Mengelers M.J., Van Gogh E.R., Kuiper H.A., Pijpers A., Verheijden J.H., Van Miert A.S. (1995). Pharmacokinetics of sulfadimethoxine and sulfamethoxazole in combination with trimethoprim after intravenous administration to healthy and pneumonic pigs. J Vet Pharmacol Ther.

[40] Ministry of Agriculture of the People’s Republic of China 2002 Announcement No. 38.

[41] Neu H.C. (1992). The crisis in antibiotic resistance. Science.

[42] Nouws J.F.M., Vree T.B., Baakman M., Driessens F., Vellenga L., Mevius D.J. (2011). Pharmacokinetics, renal clearance, tissue distribution, and residue aspects of sulphadimidine and its N4‐ acetyl metabolite in pigs. Vet Q.

[43] Passantino A., Russo C. (2008). Maximum Residue Levels of Veterinary Medicines in Relation to Food Safety: European Community Legislation and Ethical Aspects. J Verbrauch Lebensm.

[44] Sun H., Ai L., Wang F. (2007). Quantitative analysis of sulfonamide residues in natural animal casings by HPLC. Chromatographia.

[45] Van Poucke L.S.G., Van Peteghem C.H. (1994). Pharmacokinetics and Tissue Residues of Sulfathiazole and Sulfamethazine in Pigs. J Food Prot.

[46] Wang Y., Li Y., Wu J., Pei Y., Chen X., Sun Y., Hu M., Xing Y., Cao J., Li Z., Fei P., Deng R., Gu S., Hu X. (2019). Development of an immunochromatographic strip test for the rapid detection of soybean Bowman-Birk inhibitor. Food Agric Immunol.

[47] Witt J. (2006). Beitrag Nr 45: Pharmacokinetics of Sulfadiazine in Pigs.

[48] Wu X., Lin Z., Toney E., Clapham M.O., Wetzlich S.E., Davis J.L., Chen Q., Tell L.A. (2023). Pharmacokinetics, tissue residue depletion, and withdrawal interval estimations of florfenicol in goats following repeated subcutaneous administrations. Food Chem Toxicol.

